# Assessing the Utility of an Outpatient Exercise Program for Children With Cystic Fibrosis: A Quality Improvement Project

**DOI:** 10.3389/fped.2021.734292

**Published:** 2022-01-13

**Authors:** Dionne Adair, Ahmad Hider, Amy G. Filbrun, Chris Tapley, Sandra Bouma, Courtney Iwanicki, Samya Z. Nasr

**Affiliations:** ^1^Department of Pediatrics, Division of Pediatric Pulmonology, University of Michigan, Ann Arbor, MI, United States; ^2^University of Michigan Medical School, Ann Arbor, MI, United States

**Keywords:** grip strength, CF, pediatrics-children, exercise, FEV_1_

## Abstract

Children with cystic fibrosis (CF) (cwCF) suffer from inadequate weight gain, failure to thrive, and muscle weakness. The latter may be secondary to disuse atrophy (muscle wasting or reduction in muscle size associated with reduced physical activity and inflammation). Handgrip strength (HGS) is a reliable surrogate for muscle strength and lean body mass. Data from our CF center have shown an association between low HGS and forced expiratory volume in 1 s (FEV_1_) in cwCF. High-intensity interval training (HIIT) improves physical strength. Therefore, we devised a project to assess implementing a HIIT exercise program in the home setting, in order to improve physical strength in cwCF with HGS ≤ 50th percentile. Patients were instructed to complete 3–5 sessions of HIIT exercises per week. Wilcoxon matched-pairs signed-rank tests were used to compare HGS, FEV_1_, and body mass index (BMI) percentile at baseline and at a follow-up clinic visit. Follow-up was limited due to the COVID pandemic. Adherence to the HIIT regimen was poor. A total of twenty-nine cwCF participated in the program. However, a total of 13 individuals reported some form of moderate activity at follow-up and therefore constituted our final study population. There was a statistically significant increase in absolute grip strength (AGS) and FEV_1_ for these individuals. Even though the home HIIT protocol was not followed, the project demonstrated that moderate physical activity in cwCF can lead to significant improvement in HGS and overall physical strength.

## Introduction

Cystic fibrosis (CF) is a multisystemic disorder affecting more than 30,000 individuals across the United States. Due to pulmonary and gastrointestinal manifestations, people with CF (cwCF) often suffer from inadequate weight gain, failure to thrive, and muscle weakness. The latter may be secondary to disuse atrophy associated with reduced physical activity and inflammation (1). CwCF are generally weak and are less likely to participate in a vigorous activity when compared with their peers (2). Though cwCF and their families are generally aware of the benefits of physical activity, barriers such as pulmonary exacerbations and low lung function can impact their ability to regularly participate in physical activity (3).

Regular exercise improves muscle weakness in individuals with and without chronic conditions. For cwCF, physical activity augments airway clearance, an integral part of maintaining pulmonary health, and slows the rate of decline in lung function (4). Additionally, physical activity increases aerobic capacity and improves muscle strength and lean body mass (LBM) (5). LBM is positively correlated with nutritional status and also lung function in cwCF (6, 7), when compared with body mass index (BMI). Despite its ease of measurement in the clinical setting, BMI is an imperfect measurement because of its inability to differentiate between fat mass and LBM.

Handgrip strength (HGS) has been shown to be a reliable surrogate for LBM and is a practical method of assessing physical strength in the clinic setting. Data from our CF center indicate that there is a positive association between HGS and forced expiratory volume in 1 s (FEV_1_) in cwCF (8). High-intensity interval training (HIIT) improves physical fitness in children and improves the quality of life in patients with cardiometabolic disorders (5, 9, 10). HIIT exercises are offered to the individuals in our CF center during their inpatient stay for pulmonary exacerbations, in an attempt to promote muscle strengthening and augment airway clearance. We devised this project to address muscle weakness in our pediatric CF population. The goal of the project was to assess the utility of implementing a HIIT exercise program in the home setting, in order to improve physical strength.

## Method

### Study Population

CwCF ages 12–18 years, attending our Pediatric CF center, were included in this quality improvement (QI) project. This age group was identified as most likely to participate in HIIT exercises. Our physical therapist (PT) evaluated individuals at baseline to ensure that they were able to perform HIIT exercises. Individuals with HGS of ≤50th percentile for age who were able to perform HIIT exercises were offered the intervention of an individualized HIIT home training program. Individuals included for final analysis met criteria of HGS ≤ 50th percentile and had returned for serial grip strength measurements (see Supplementary Figure 1). This manuscript originated from data from a QI project in our center.

### Project Design and Measurement

**Figure 1:**
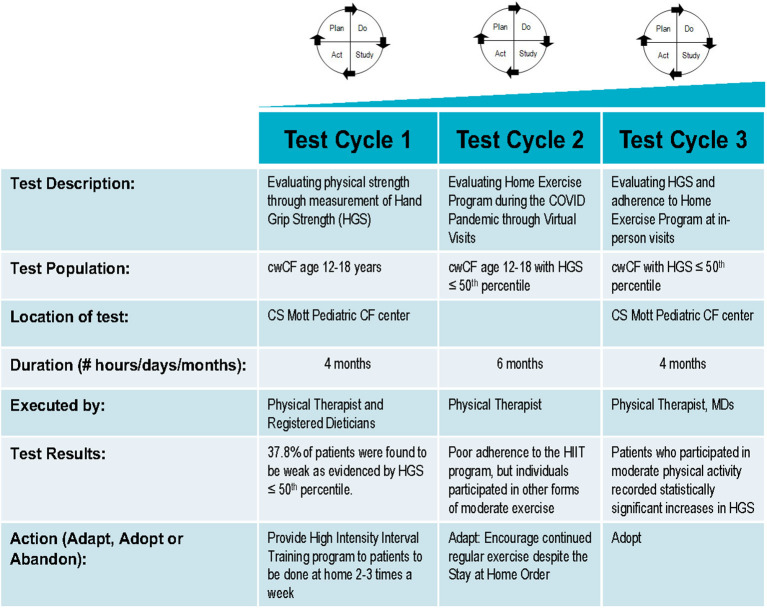
Plan-Do-Study-Act (PDSA) ramp describing test cycles 1 to 3 of the home exercise program.

The QI project was reviewed by our Medical School Institutional Review Board (IRBMED), and it was determined that it does not require institutional review board (IRB) approval because it does not satisfy the definition of research under 45 CFR 46.102(l) and 21 CFR 56.102(c). The project was undertaken in a series of Plan-Do-Study-Act (PDSA) cycles (Figure 1). The first PDSA cycle involved identifying cwCF aged 12–18 years with HGS ≤ 50th percentile and offering them a HIIT exercise program to be done at home. Trained personnel measured each participant's grip strength with a calibrated Jamar Plus digital hand dynamometer (Patterson Medical, Warrenville, IL, USA) using the American Society of Hand Therapists' measurement protocol. For the measurement, individuals were seated with their shoulders adducted, elbow flexed at 90°, and forearms in a neutral position. The handle was positioned such that the individuals were able to wrap their thumb around one side of the handle and their fingers around the other side, with their intermediate phalanges covering the face of the handle and without the tips of their fingers coming into contact with the palm of their hand. Three measurements were taken on each hand, alternating between hands with a 10- and 15-s break between each measurement. Individuals were encouraged to squeeze harder until the number on the digital read-out stopped rising (11). Age- and gender-specific percentiles for absolute grip strength (AGS) were determined from the pre-published percentile charts, originating from our center, that were derived from data collected by two survey cycles of the National Health and Nutrition Examination Survey (8).

HIIT is a training technique involving periods of high-intensity exercises alternating with low-intensity exercises or rest (12). The HIIT home training program offered to the project participants was individualized and included 5 min of a warm-up and 16–24 min of HIIT exercises followed by stretching, to be done 3–5 times per week. Individuals were allowed to choose 8 exercises from a list of exercises that are considered high intensity, including jumping jacks, burpees, and squats. Each exercise was followed by a rest period of 1–2 min until the patient returns to baseline work of breathing as assessed by the 15 count breathlessness scale. Verbal and written instructions were provided during the initial clinic visit by our PT, who was then available for consultation by phone between visits.

The second PDSA cycle involved follow-up of the individuals who were offered the home exercise program. This was done by our PT. The type and frequency of activity that individuals were involved in were recorded. If individuals did not report completing the HIIT training, they were questioned for any additional activity or exercise they were completing. The exercise program was then adapted for patients participating in non-HIIT activity, to allow them to complete exercises of their choice. This was done in order to encourage patients to remain active. All reported activities were classified according to metabolic equivalents (MET) values listed in the Youth Compendium of Physical Activities (13) and defined as very light/light (<3.0 METs), moderate (3.0–5.9 METs), or vigorous (≥6.0 METs) activities. If an individual reported no activity, barriers were addressed, and additional education was provided.

In the third and final PDSA cycle, we evaluated adherence to the exercise program and repeated HGS measurements in those individuals able to return for an in-person visit. In our center, individuals are followed up every 3 months. Initially, we planned to obtain HGS measurements at these quarterly visits; however, with the onset of the coronavirus pandemic, many in-person clinic visits were converted to virtual visits. Therefore, we obtained grip strengths when individuals and/or staff were available during an in-person encounter. Follow-up HGS measurements were then compared with baseline measurements. The medical record was also reviewed to document interval change in spirometry, BMI, and BMI percentile.

### Statistical Analysis

Wilcoxon matched-pairs signed-rank test was used to compare HGS, lung function, BMI, and BMI percentile at baseline and follow-up assessments of the entire cohort. A multiple linear regression model was used to determine independent predictors of increased HGS. For all comparisons tests, a *p*-value of < 0.05 was considered statistically significant. R version 4.0.5 (14) was the statistical software used for analysis.

## Results

One hundred three cwCF between the ages of 12 and 18 had HGS assessed over a 4-month period. Of those tested, 39 (37.8%) were found to have HGS ≤ 50th percentile. Twenty-nine of the 40 children returned for follow-up measurement. The median time to follow-up was 8 (2–10) months (Table 1), due to delayed in-person follow-up because of the pandemic. The median AGS was 24 kg [11.8–45.8] with the median AGS percentile at the 10th percentile. In contrast to AGS, the median BMI and BMI percentile were within the normal range at 21.3 kg/m^2^ and 68th percentile, respectively.

**Table 1:** Baseline characteristics of children with CF with HGS ≤ 50th percentile (*N* = 29).

**Baseline characteristic**	**Median (range)**
Age (years)	15.99 (12.04–18.87)
Time to follow-up (months)	8 (2–10)
BMI (kg/m^2^)	21.37 (17.56–31.29)
BMI percentile	68.16 (19.73–98.79)
Maximum AGS (kg)	24 (11.8–45.8)
AGS percentile	10 (4–50)
FEV_1_ (percent predicted)	85 (47–104)
	***N*** **(percent)**
Individuals on highly effective modulators	21 (72)
Individuals on at least moderate activity	9 (31)

*AGS, absolute grip strength; BMI, body mass index; FEV_1_, forced expiratory volume in 1 s*.

The initiation of the project coincided with the approval of elexacaftor/tezacaftor/ivacaftor (ELX/TEZ/IVA), a highly effective modulator for individuals with CF at 12 years of age and older. Sixty-three percent of our cwCF had been on ELX/TEZ/IVA at the time of the baseline measurement. Four children taking tezacaftor–ivacaftor at enrollment changed to ELX/TEZ/IVA after the baseline assessment. These 4 individuals were excluded from the analysis to eliminate possible confounding factors.

Of the 29 children with serial grip strength measurements, only 2 reported participating in the HIIT program, with one discontinuing after a month due to the perceived difficulty of the exercises. Thirteen (44.8%) of the 29 individuals with HGS data had information on participation in an activity at baseline and follow-up and had been on ELX/TEZ/IVA at baseline. These 13 individuals constituted our final study group. Within this group, there was an 11.2% increase in median AGS and a 100% increase in median AGS percentile at visit 2 (Table 2). There was a small increase in median BMI and BMI percentile. We also looked at lung function, which was assessed at every follow-up visit. There was an increase in FEV_1_ of 11.7%, which was statistically significant.

**Table 2:** Comparison between Visit 1 (baseline) and visit 2 (follow-up) in children with CF with HGS ≤ 50th percentile (*N* = 13).

	**Visit 1**	**Visit 2**	**Percent change**	***p*-value**
AGS (kg)	24.1 (11.8–45.8)	26.7 (19–49.8)	11.25	<0.001
AGS percentile	10 (4–50)	22.2 (4–70)	100	<0.001
Absolute BMI (kg/m^2^)	21.37 (17.56–31.29)	22.25 (17.26–31.56)	3.88	0.04
BMI percentile	68.16 (19.73–98.79)	70.84 (21.71–98.77)	3.93	0.98
FEV_1_ (% predicted)	85 (47–107)	95 (52–123)	11.76	0.006

*Parameters for Visits 1 and 2 are represented as median (range). AGS, absolute grip strength; BMI, body mass index; FEV_1_, forced expiratory volume in 1 s; CF, cystic fibrosis; HGS, hand grip strength*.

We then divided this cohort of 13 patients into two groups: one with stable activity (n of 7) and one with increased activity (n of 6). The group with stable activity was already participating in at least moderate activity at baseline and continued to participate in a similar activity at follow-up. The group with increased activity included children who were inactive at baseline but reported at least moderate activity at follow-up. Though both groups had increases in AGS [median percent change 6.8% for the stable group vs. 10.9% for the increased group], there was no significant difference between the two groups in the baseline-to-follow-up percent change in grip strength or grip strength percentile. In addition, there was no significant difference in the percent change in BMI or lung function between the groups. To determine factors associated with percent change in HGS, we analyzed the data using a multiple linear regression model with the percentage change in HGS and percentage change in HGS percentile as the dependent variables and change in BMI, time to follow-up, female gender, and increased activity as independent variables. From this model, there was a significant association between female gender and lower average percent change in AGS percentile (*p* < 0.01). Though the average percent change in AGS and AGS percentile in the increased activity group was positive in the model, this was not statistically significant.

## Discussion

Physical strength is decreased in cwCF for a variety of reasons including poor nutritional status and decreased physical activity. HGS is an indicator of physical fitness and general muscle strength in children and adults (15). It is also a more practical method of assessing LBM. When compared with BMI, LBM does not incorporate fat mass; therefore, it better correlates with nutritional status and lung function in cwCF (16). Data from our CF center indicate a positive association between HGS and lung function in children with CF (8).

We first set out to identify children in our CF center who were weaker, as evidenced by low AGS. Almost 40% of children and adolescents had HGS ≤ 50th percentile for age. Interestingly, their average BMI was within normal limits. This is in keeping with prior studies that demonstrated the inadequacy of BMI in assessing body composition and nutritional status. In a pilot study done in children with CF who were hospitalized, Gibson et al. found that mean HGS z-scores were very low compared with the standard, whereas mean BMI z-scores were much closer to the standard (16). Similarly, among 201 medically stable CF individuals aged 6–21 years in our pediatric center, 40.7% of individuals with a BMI ≥ 50th percentile were weak for their size as evidenced by an HGS measurement < 25th percentile (8). Therefore, HGS may be more sensitive in detecting early changes in muscle mass than BMI.

In an attempt to improve physical strength in those children with low HGS, we devised a HIIT workout plan. HIIT increases LBM in healthy adults. It has similar effects on increasing fitness as a moderate-intensity aerobic training and in a shorter time. Therefore, time-efficient HIIT workouts may be more appealing to cwCF. Brown et al. used a 12-week program of 3 sessions and found that their intervention of HIIT exercises resulted in increased LBM and reduction in total body fat percentage in healthy young adult females (5). Smaller studies and case reports in cwCF have shown HIIT exercises to be safe and effective in improving exercise capacity and in a shorter time than standard exercise programs (17, 18). Verbal and/or written guidelines on exercise have been shown to be effective in increasing self-reported exercise activity in children with CF. A randomized trial is presently underway in CF adolescents and adults, which may give us insight into the efficacy of the web-based intervention in promoting physical activity (19).

We employed a combination regimen including verbal instruction by our PT, who also provided handouts with exercises that could be incorporated into a HIIT regimen. Though follow-up was severely affected by the pandemic, which led to decreased patient retention and longer time to follow-up, 6/29 (20%) of eligible children were motivated to start a moderate exercise program on their own (increased activity group). Only 2 children participated in the HIIT program. The remaining individuals chose different aerobic activities, which included swimming, cheerleading, weightlifting, walking, and running. Competing priorities, degree of difficulty, and lack of interest in the HIIT program were identified as barriers to participation in the program. Twenty-four percent (7/29) who were already participating in at least moderate activity at baseline (stable activity group) continued to participate in aerobic activities of their choice at their follow-up visit. Following the HGS measurement, the explanation of the benefits of exercise, and the encouragement of cwCF to exercise by the PT, both groups demonstrated significant improvement in AGS, AGS percentile, and FEV_1_ percent predicted. This would suggest that even in patients who are active at baseline, there is a potential of continuing to increase physical strength and lung function. With the knowledge that the increased activity group had a higher percentage change in AGS, we created a multiple linear regression model to ascertain predictors for percentage change in grip strength. After change in BMI, time to follow-up, and gender were controlled, the average difference in AGS was positive but not statistically significant. This may have been due to our small cohort. Given that females are generally weaker than their age-matched male counterparts secondary to reduced muscle mass, it was not surprising that the female gender had a much lower average percent change in the AGS percentile in the model.

Though HGS increases with age, the absolute increases in values in our cohort exceeded that which would have been expected throughout the project. More frequent follow-up may have further improved/encouraged other children to continue or start an activity. In addition, it was clear that individuals either continued to participate in an exercise of their choosing or commenced aerobic exercises other than the HIIT regimen that was presented to them. This suggests that the message to cwCF is that implementing home exercise is important and that any activity (at least moderate in nature) would be adequate in improving their muscle strength and potentially their lung function. Therefore, emphasis should be placed on encouraging moderately intense physical activity at regular intervals regardless of its nature.

The Cystic Fibrosis Foundation recommends that cwCF maintain a BMI of at least the 50th centile. This has been positively associated with better nutritional status and lung function. Similarly, better LBM is associated with better lung function and may correlate even more than BMI (6). Ionescu et al. found that cwCF with low fat-free mass (FFM) (which is approximately equal to LBM) had a lower mean FEV_1_% predicted compared with the normal FFM individuals (20). A recent publication from our CF center revealed a statistically significant association between HGS and percent predicted FEV_1_ across all BMI percentiles (8). Hence, by increasing HGS and therefore increasing LBM through physical activity, an individual may see positive effects on lung function. It is hypothesized that this improved lung function is due to increased respiratory muscle strength associated with optimizing overall LBM (21). In our cohort, there was a statistically significant change in percent predicted FEV_1_ at follow-up.

Despite the significant improvement seen with the HGS, we recognize that our QI project had some limitations. Our cohort was limited by the small number of children who qualified for this program. Additionally, the majority of the children included in this QI initiative were started on the highly effective modulator ELX/TEZ/IVA within 3 months of beginning the project. This presents a possible confounder; however, we eliminated the children who started ELX/TEZ/IVA after baseline grip strength assessment. Also, since we did not analyze patients who participated in very light/light activities (<3.0 MET), we could not comment on whether lower-intensity exercises could also lead to changes in AGS improvement. Furthermore, our cohort excluded patients with HGS > 50th centile; therefore, we cannot comment on whether stronger patients can also increase their HGS or whether their participation in exercise could have accounted for higher HGS measurements. A longer follow-up period would have allowed us to look at the sustainability of moderate exercise on improving HGS and lung function in children with CF. However, there have been studies in children with CF which demonstrate the feasibility of long-term aerobic training programs of up to 3 years (22).

## Conclusion

A significant number of cwCF are weaker than their age-matched peers. Regular physical activity involving at least moderate aerobic exercise can increase HGS and LBM which may, in turn, have positive effects on physical fitness, lung function, and overall quality of life in this population.

## Data Availability Statement

The raw data supporting the conclusions of this article will be made available by the authors, without undue reservation.

## Author Contributions

DA, CT, CI, and SB contributed to the acquisition of the data. AH contributed to the analysis and interpretation of the data. DA, AF, and SN contributed to the interpretation of the data. DA wrote the first draft of the manuscript. All authors contributed to the conception and design of the project, manuscript revision, and read and approved the submitted version.

## Funding

Funding was provided through Dr. Nasr's discretionary.

## Conflict of Interest

SB served as a speaker for Abbott Nutrition and Health Institute. The remaining authors declare that the research was conducted in the absence of any commercial or financial relationships that could be construed as a potential conflict of interest.

## Publisher's Note

All claims expressed in this article are solely those of the authors and do not necessarily represent those of their affiliated organizations, or those of the publisher, the editors and the reviewers. Any product that may be evaluated in this article, or claim that may be made by its manufacturer, is not guaranteed or endorsed by the publisher.
