# Supplementary material for: Assessing the Utility of an Outpatient Exercise Program for Children With Cystic Fibrosis: A Quality Improvement Project
Source: Front Pediatr. 2022 Jan 13;9:734292. doi: 10.3389/fped.2021.734292 (PMC8793844; doi:10.3389/fped.2021.734292)
Supplement: Supplementary file 1 [file Data_Sheet_1.docx]

Supplementary Material

# Supplement

PRISMA Flow Diagram for Individuals included in the Project

CwCF age 12-18 years with HGS measured between 11/2019 to 3/2020.

(n = 103)

**Initial HGS measurement**

Individuals with baseline HGS ≤ 50^th^  percentile for age

(n=39)

**Return HGS measurements**

**Individuals included in analysis**

Patient’s excluded due to not returning for serial measurements

(n=10)

Individuals who returned for serial HGS measurements & included in final analysis

(n=29)

CwCF, children with CF; HGS, hand grip strength
